# Protein-Based Nanoparticles for the Imaging and Treatment of Solid Tumors: The Case of Ferritin Nanocages, a Narrative Review

**DOI:** 10.3390/pharmaceutics13122000

**Published:** 2021-11-25

**Authors:** Francesco Mainini, Arianna Bonizzi, Marta Sevieri, Leopoldo Sitia, Marta Truffi, Fabio Corsi, Serena Mazzucchelli

**Affiliations:** 1 Dipartimento di Scienze Biomediche e Cliniche “L. Sacco”, Università di Milano, 20157 Milano, Italy; francesco.mainini@unimi.it (F.M.); arianna.bonizzi@unimi.it (A.B.); marta.sevieri@unimi.it (M.S.); leopoldo.sitia@unimi.it (L.S.); 2 Istituti Clinici Scientifici Maugeri IRCCS, 27100 Pavia, Italy; marta.truffi@icsmaugeri.it

**Keywords:** ferritin, cancer, tumor targeting, drug delivery, imaging

## Abstract

Protein nanocages have been studied extensively, due to their unique architecture, exceptional biocompatibility and highly customization capabilities. In particular, ferritin nanocages (FNs) have been employed for the delivery of a vast array of molecules, ranging from chemotherapeutics to imaging agents, among others. One of the main favorable characteristics of FNs is their intrinsic targeting efficiency toward the Transferrin Receptor 1, which is overexpressed in many tumors. Furthermore, genetic manipulation can be employed to introduce novel variants that are able to improve the loading capacity, targeting capabilities and bio-availability of this versatile drug delivery system. In this review, we discuss the main characteristics of FN and the most recent applications of this promising nanotechnology in the field of oncology with a particular emphasis on the imaging and treatment of solid tumors.

## 1. Introduction

Nanoparticle-based drug delivery systems have the capacity to enhance the physicochemical properties of a wide variety of drugs used in oncology to limit off-site side effects and improve their therapeutic efficacy [1,2,3]. The ideal nanocarrier should be bio-compatible and be able to avoid recognition by the reticuloendothelial system (RES), composed of tissue-resident macrophages and phagocytes in the bloodstream, capable of efficiently clearing exogenous nanoparticles (NP) from the circulation [4]. Natural proteins nanocages have a distinctive advantage in this regard, in comparison to synthetic NP (liposomes, polymeric NP, micelles and dendrimers), since they are virtually invisible to the immune system and display great biocompatibility coupled with minimal toxicity. An exception is represented by virus-like particles (VLPs), which are also composed of self-assembled proteins that are, in some cases, highly immunogenic [5].

**Figure 1:**
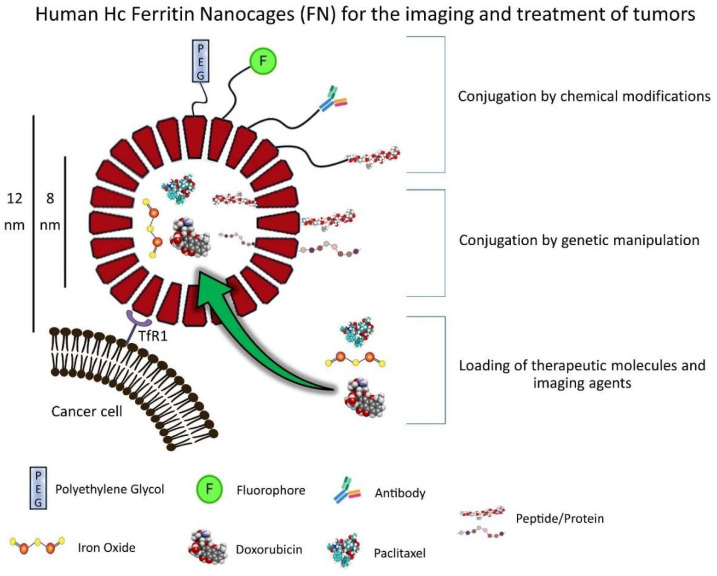
FN as a protein-based delivery system for oncological therapeutics and imaging agents. FNs are composed of 24 Hc subunits that can be chemically or genetically modified to couple a large variety of molecules (antibodies, peptides, fluorophores, polyethylene glycol (PEG) and others) to their surface (N-terminus) or internal cavity (C-terminus). Furthermore, FNs can be loaded with different drugs and imaging agents and have intrinsic targeting capabilities toward the receptor TfR1, which is overexpressed in many tumors. This cartoon was created by using BioRender (https://biorender.com/, accessed on 2 November 2021).

**Figure 2:**
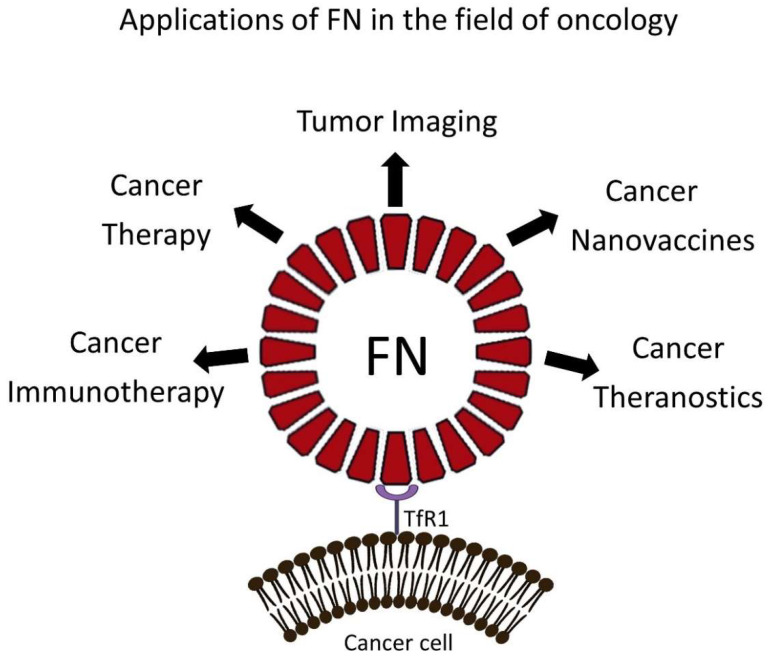
Applications of FNs in the field of oncology. FNs are versatile drug delivery systems. They can be loaded simultaneously with anticancer and imaging agents to provide effective antitumor therapy that can be monitored by different imaging modalities. In addition, FNs can be loaded with immunomodulatory drugs to remodel the TME or can be developed to incorporate tumor associated antigens to induce specific adaptive immune responses against cancer cells, in the case of nanovaccines. This cartoon was created by using BioRender (https://biorender.com/, accessed on 2 November 2021).

Endogenous self-assembled NPs can be synthesized by many cell types and are primarily used to store and/or distribute to different tissues a wide variety of molecules, such as nutrients and biochemical signals. NPs of this kind are quite diverse in terms of size and physiological activity. Some examples are ferritin nanocages (FNs), heat-shock protein cages, vault ribonucleoparticles, the E2 protein of the pyruvate dehydrogenase multienzyme complex, chaperones, carboxysomes and other enzyme complexes [6]. Unfortunately, most of these protein-based NP are understudied and have, so far, limited applications in the field of oncology. However, FNs have been studied extensively due to their intrinsic targeting capabilities toward the Transferrin Receptor 1 (TfR1), which is highly expressed in many tumors, making them very appealing for drug-delivery applications in oncology. Furthermore, the small size and high customization potential make them ideal candidates for the development of novel nanomedicines able to deliver a wide variety of drugs to the tumor microenvironment (TME). This review describes the structure and function of FN, modifications of the nanocages by chemical or genetic manipulation (Figure 1) and novel applications of this nanotechnology for the imaging and treatment of solid tumors (Figure 2).

## 2. FN Structure and Properties

Ferritin self-assembles in hollow icosahedral-shaped nanocages with inner and outer dimensions of 8 and 12 nm, respectively [7]. In mammalian cells, ferritin is composed of heavy-chain (Hc, 21 kDa) and light-chain (Lc, 19 kDa) subunits (24 in total between the two) which are structurally similar. FN employed as a delivery device in cancer application are mostly constituted only by Hc subunits of human ferritin. Ferritin and FNs are remarkably stable in biological fluids and are resistant to denaturants, including high temperatures (>80 °C) [8]. Each subunit is composed of four long helixes, a short helix and a long loop [9]. The C-terminal of each subunit folds into the inner cavity, while the N-terminal is exposed on the outer surface of the nanocage. The ratio between Hc and Lc subunits is determined by ferritin’s primary role in tissues. For example, in the hearth and brain, the Hc is more abundant, while, in the liver and spleen, the Lc is predominant. The Hc subunit contains a dinuclear ferroxidase site that is located within the four-helix bundle, while the Lc provides efficient sites for iron nucleation and mineralization [10]. Ferritin and FN carry six C4 channels and eight C3 channels. The C3 channels have hydrophilic properties and allow the passage of Fe(II) ions and water molecules in and out of the protein cage. On the other hand, the C4 channels allow the passage of small hydrophobic molecules [8].

Ferritin in the bloodstream is mainly composed of Lc subunits [8], which seem to be secreted primarily by macrophages [11]; however, their role in the serum is still highly debated. Nonetheless, high ferritin levels have been linked with ongoing infections and chronic inflammation, while its reduced levels have been correlated with iron deficiency [12,13,14]. Interestingly, it can be localized in cells both in the cytoplasm and in the nucleus. Iron stored in ferritin can be utilized by the cell in a process mediated by autophagy, where it is transported to the lysosomes and iron is released in a pH-dependent manner [15]. On the other hand, in conditions of oxidative stress, ferritin can convert DNA damaging Fe(II) to harmless Fe(III), thus limiting DNA damage mediated by the formation of hydroxyl radicals through the Fenton reaction [16,17,18]. Two proteins, poly(rC)–binding protein 1 (PCBP1) and nuclear receptor coactivator 4 (NCOA4), are involved in the transport of iron inside and outside ferritin [19]. Furthermore, it has been proposed that O-glycosylation of the Hc could be involved in the nuclear translocation of Ferritin, which maintains its intact structure during this process [20,21]. FNs share all structural features and properties with their physiological form, and they often have been demonstrated to be managed by the cells and the tissue as natural ferritin [21,22].

### Strategies for Loading FN

**Figure 3:**
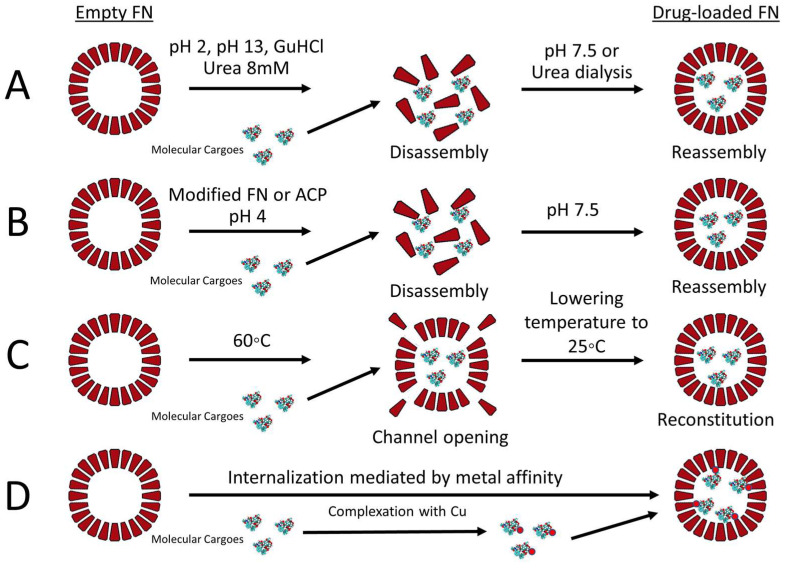
Biochemical strategies used to load different cargoes into FNs: (**A**) pH or urea-mediated disassembly–reassembly methodology; (**B**) modified ferritins or ACP can be utilized to disassemble FN at pH 4, and then pH 7.5 is used to reassemble FN; (**C**) high temperatures partially destabilize FN to allow channel openings with consequent drug loading, and then lowering the temperature can slowly reconstitute the natural conformation of FN; (**D**) molecular cargoes can be complexed with Cu(II) or other metal ions which have a high affinity for the internal cavity of FN. This methodology permits the loading of hydrophobic molecules with some limitations.

Molecular cargoes can be loaded into the inner core of FN by different methodologies (Figure 3). Extreme pH (2 or 13) is used to transiently disassemble the protein nanocage into monomers that can reassemble by adjusting the pH toward neutrality. By employing this methodology, FN can be loaded with different chemotherapeutic drugs. Interestingly, only minor differences in the loading efficiency between doxorubicin (DOX), epirubicin (EPI), daunorubicin (DAU) and idarubicin (IDA) were seen, despite their differences in terms of hydrophobicity [23]. In addition, high concentrations of guanidine hydrochloride (GuHCl) or urea are able to disrupt the non-covalent forces which support FNs’ structure, leading to their disassembly. This process can be reversed by dialysis to remove the excess of chaotropic agents, leading to the recovery of the original nanostructure with consequent loading of molecular cargoes in the inner cavity [24]. More recently, atmospheric cold plasma (ACP) technology was implemented to reduce the α-helix/β-sheet contents and thermal stability of FN to allow disassembly at a pH of 4. This technique can be utilized to load molecules which are susceptible to extreme pH conditions and could be degraded during the loading procedure [25]. In addition, our group has showed that the loading of molecules sensitive to low pH can be achieved during the reassembly phase by adding the molecule of interest to the ferritin-containing solution after the adjustment of the pH toward neutrality [26]. In another report, Jiang and colleagues developed a methodology that is able to provide high loading of DOX and high recovery of FN by incubating DOX with FN at 60 °C for 4 h [27]. This loading methodology enables the opening of FN’s channel to introduce DOX without disrupting FN’s structure. Lastly, by taking advantage of the natural capacity of FNs to encapsulate iron in their cavity, several metal ions can be coupled to molecules of interest that can then be loaded into FNs. In this case, the final loading efficiency of the chosen drug depends on its binding affinity for the metal ion, the FN species used and preparation conditions. For example, Zhen and colleagues suggested that, between Cu(II), Mn(II), Zn(II) and Fe(III) metal ions, the use of copper resulted in the highest loading rate of DOX into FN [28]. A more detailed comparison of loading methodologies for DOX into FN has been reviewed by He and colleagues [29], while Zhang and colleagues provide specific protocols regarding the loading of various drugs into FNs by using different methodologies [30].

Overall, FNs’ physicochemical properties (small size and negative Z potential), together with their intrinsic capacity to avoid recognition by RES and targeting capability toward TfR1-expressing tumor cells, make them an ideal candidate for the development of drug delivery systems for nanobiotechnological applications in the field of oncology (Table 1). Moreover, drug loading and targeting efficiency could be enhanced by chemical and genetic manipulations of FNs, as is discussed in the following section.

## 3. Production and Modifications of FN

FNs utilized in preclinical studies are usually produced as recombinant protein in *E. coli* strains engineered to express only the human Hc subunit. This procedure involves the transformation of bacteria with a plasmid containing the Hc sequence of interest, which is then purified by anion-exchanger columns after treatment at 70 °C. The resulting FNs are composed, in this case, of 100% Hc subunits [66]. Otherwise, FNs can be purified from the horse spleen, where the ratio between Hc and Lc subunits was found to be ~1/10 [67]. To ensure that purified FNs are not contaminated by endotoxins that could impact both in vitro and in vivo experiments, additional procedures to remove endotoxins might be required [68].

The genetic manipulation of the Hc-FN DNA sequence led to the development of more than one hundred variants to introduce novel functionalities that are able to improve the drug loading, biodistribution and targeting properties of FNs [69]. For example, the self-assembly properties of FNs can be altered to produce novel nanostructures comprising 8 or 48 subunits instead of 24 [70,71]. In addition, ferritin can be modified to produce nanocages that can disassemble at a pH of 4 or 6, instead of 2 [72,73,74]. Intriguingly, Gu and colleagues developed His-modified ferritins that do not self-assemble at neutral pH. However, metal ions or a pH of 10 induce self-assembly with consequent increases of the drug-loading efficiency, as compared to the standard pH methodology discussed previously [75]. Unfortunately, the stability in serum of His-modified ferritins was not evaluated, and it is unclear if these nanoconstructs are suitable for in vivo studies.

Different strategies have been recently employed to enhance the half-life of FNs in the circulation to provide higher tumor accumulation and reduce clearance by RES. For example, Wang and colleagues developed a novel FN that includes an albumin-binding domain that is able to increase FNs’ half-life by 17 times, as compared to the standard FN [76]. In another report, an amino acid sequence rich in proline (P), serine (S) and alanine (A) residues (PAS polypeptide) was inserted by genetic manipulation into FN to increase blood half-life and DOX encapsulation efficiency [77,78]. Interestingly, the insertion of two glutamate residues in the PAS sequence (PASE) further improved FNs’ accumulation to the tumor site [79]. In another report, Jin and colleagues introduced in the ferritin construct a blood circulation prolonging (BCP) peptide derived from the phage M13. The generated FN (BCP1–FN) showed improved circulation time compared to standard FN (20 h compared to 2 h). In addition, when loaded with DOX, BCP1–FN–DOX showed superior therapeutic efficacy in a mouse model of melanoma compared to FN–DOX and free DOX. Intriguingly, the authors suggested that the RGD portion of the BCP1 peptide could be responsible for the binding of BCP1–FN to peripheral blood cells, particularly platelets, which are able to protect the nanocages from RES recognition [31]. Nonetheless, blood cells’ “hitchhiking” has recently emerged as one of the strategies that can be employed to enhance the delivery of NP to the tumor site [80,81].

Since FNs are composed of different subunits, novel FN-based nanostructures have been developed with combinations of different ferritins resulting in hybrid nanocages with interesting physicochemical properties. Ahn and colleague developed a hybrid FN composed of modified subunits (F160) and standard Hc in a ratio 1:1. F160 was devised to provide large pores to FN and was produced by removing the C-terminal channel forming E-helix from the Hc sequence. The resulting hybrid FN (nicked–FN) allows for the encapsulation of DOX by simple incubation, improving the loading of DOX and the recovery of the nicked–FN–DOX in comparison to encapsulation in unmodified FN or with the pH-mediated disassembly and reassembly methodology [82]. Another strategy to enhance DOX loading into FN was developed by producing a mutant FN that displays an enhanced affinity for copper ions [32]. In another report, FNs were modified with the addition of biotin accepted peptide, which resulted in biotinylated FN that can be more easily modified by the addition of streptavidin-tagged molecules [83]. These modified FN could be used in a variety of immunoassays based on streptavidin-tagged antibodies to increase the sensitivity. It is unsure if they can be employed for in vivo studies.

The delivery of nucleic acids by NP-based delivery systems has always been a primary goal of the research effort in the field of nanotechnology. Interestingly, modified FNs with the addition of a cationic polypeptide were developed to facilitate the incorporation of siRNAs in FNs’ nanostructure [33,84]. However, it has also recently been shown that unmodified FNs could incorporate siRNAs by pH-mediated disassembly and reassembly methodology [85].

FNs can also be modified to include immunogenic peptides that are able to induce immune responses against specific antigens. As proof of principle, Kanekiyo and colleagues developed a nanovaccine against the H1N1 virus based of FNs that were modified to include the viral hemagglutinin sequence. Preclinical testing of the developed nanoformulation showed induced protection of animal models to H1N1 infection [86]. More recently, FN-based anticancer nanovaccines have been developed and were tested successfully in preclinical models [56,87,88].

Interestingly, many FN variants have been developed to include novel targeting ligands. For example, Jiang and colleagues introduced, by genetic manipulation, a hepatocellular carcinoma (HCC)-targeting peptide to the FN’s structure that was then loaded with DOX. This novel formulation showed superior activity compared to free DOX in reducing HCC tumor growth and metastases in preclinical models [48,89]. In another report, the PD-L1 binding peptide 1 (PD-L1pep1, CLQKTPKQC) was introduced into ferritin’s sequence to generate an FN targeted to PD-L1 [34]. Another well-studied tumor-targeting ligand is the tLyp-1 peptide, which binds the receptor Neuropilin 1 expressed in the stroma of many types of tumors [90]. Modified FNs were developed to include the tLyp-1 peptide in the external structure of the nanocage and were subsequently loaded with Paclitaxel (PTX). The resulting FNs (tLyp–FN–PTXs) showed enhanced uptake by tumor cells and were able to control tumor growth in vivo compared to free-PTX or FN, where the sequence of tLyp was mutated (m-tLyp–FN–PTX) [35].

Beyond genetic manipulation, FNs have available primary amines on their surface that can be exploited for chemical conjugation purposes. N-hydroxysuccinimide (NHS) ester or maleimide groups, in combination with 1-ethyl-3-(-3-dimethylaminopropyl) carbodiimide (EDC), are often used to couple peptides, PEG, fluorophores or antibodies to FN in a buffered solution, without the use of organic solvents [42,91]. This coupling methodology is often employed to develop fluorescent versions of FNs that can be utilized in a variety of in vitro and in vivo assays, including flow cytometry, fluorescence microscopy and live imaging, which are critical techniques for NP characterization and for the evaluation of biodistribution and targeting capacity of novel formulations of FNs. In a recent report, FNs were modified with the addition of positively charged polyamine dendrimers (PAMAM) to allow efficient loading of nucleic acids. MiRNA-loaded FNs were successfully used to target leukemia cells and showed promising in vitro results [92].

Overall, both genetic and chemical manipulations can enhance multiple aspects of the intrinsic properties of FNs, such as targeting, loading and half-life. However, it is unknown if these modifications could induce the production of specific anti-ferritin antibodies when administered in humans. Interestingly, it has been shown that modifications such as PEGylation could result in the generation of anti-PEG antibodies [93]. Therefore, it is plausible that some of the developed modifications of the native human Hc subunit, by both genetic and chemical manipulation, could potentially reduce the effectiveness of FN after multiple administrations, limit their targeting capabilities and induce undesirable immunogenic reactions [93]. Furthermore, the FN’s origin could be an important factor contributing to immunological side effect. These potential complications should be carefully taken in consideration to ensure the success of modified FNs in the prospect of clinical translation [94].

## 4. FN-Based NPs for Cancer Treatment in Preclinical Models

One of the main issues in the delivery of chemotherapeutics for cancer treatment is the onset of off-site side effects, which can cause a wide spectrum of complications, such as infections, neuropathies, cytopenias, nephrotoxicity, cardiotoxicity and hepatotoxicity [95,96,97,98]. NP-based delivery systems are utilized in oncology primarily to reduce the severity of these side effects, improving drug accumulation at the tumor site. Examples of nanotherapeutics currently used in the clinical practice are Doxil™, Abraxane™, Marqibo™ and DaunoXome™, which are NP-based platforms for the delivery of DOX, PTX, Vincristine and DAU, respectively [1].

Interestingly, FNs have been extensively studied as nanocarriers for DOX, since this hydrophilic drug can be encapsulated efficiently into FNs and can be delivered to tumor cells by the TfR1-mediated intrinsic targeting capabilities of FNs. Our group and others have shown that not only are FN–DOX formulations superior to free DOX or Doxil™ in controlling tumor burden in preclinical models of cancer, but they also dramatically reduced drug cardiotoxic effects, as compared to the free DOX [48,99,100,101]. In another report, Huang and colleagues developed a hybrid FN–DOX formulation for the treatment of lung cancer. It is composed of PEGylated Hc subunits to provide stealth capabilities and non-PEGylated Hc subunits to allow the binding of the nanocage to TfR1. In vivo results showed that, after intratracheal administration of hybrid FN–DOX, the tumor burden in a orthotopic murine model of lung cancer (3LL) was dramatically reduced when compared to free DOX [37]. Apart from DOX, platinum-based chemotherapeutics (cisplatin, oxaliplatin, Pt(II) terpyridine and carboplatin) have been successfully encapsulated in FNs and have shown encouraging antitumor activity in preclinical models of cancer [53,102,103]. Recently, Ferraro and colleagues developed a novel FN loaded with Arsenoplatin-1 (Pt(µ-NHC(CH_3_)O)_2_ClAs(OH)_2_), which combines the cytotoxic effects of both cisplatin and arsenic trioxide. Preliminary in vitro results showed that this novel formulation provides selectivity toward cancer cells, but, unfortunately, it was not tested in vivo [50].

**Figure 4:**
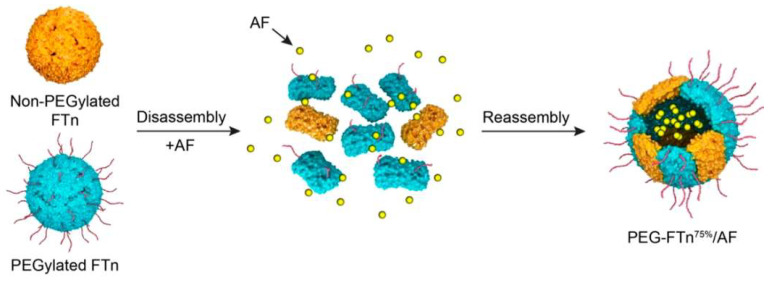
Structure and loading procedure for AF-loaded hybrid PEGylated FN. Hybrid FNs can be developed by utilizing the pH disassembly/reassembly methodology, starting from two different FNs (in this case, a PEGylated FN and non-PEGylated FN). In addition, prior to the reassembly phase, anticancer drugs can be added, resulting in their inclusion inside the FN nanostructure after reassembly. Adapted from [39], American Chemical Society, 2019.

The development of drug resistance often occurs after treatment with standard chemotherapeutics, and it can be mediated by the activity of the transporter multidrug resistance protein 1 (MDR1), which is upregulated in the hypoxic areas of tumors and facilitates the excretion of chemotherapeutics outside the tumor cell membrane [104]. Interestingly, hypoxia in the TME can induce the expression of TfR1 mediated by hypoxia-inducible factor-1α (HIF-1α) in tumor cells [105]. Hence, FN-based NPs could be employed to specifically target hypoxic areas in tumors. For this purpose, Huang and colleagues developed a hybrid FN (composed of 75% PEGylated subunits) (Figure 4) for the delivery of the HIF-1α inhibitor Acriflavine(AF). This nanoformulation was particularly effective when used in combination with cisplatin, since the delivery of AF to the TME was able to reduce the expression of MDR1 on tumor cells, thus reducing the development of resistance to cisplatin, which was not effective as standalone treatment in the 3LL lung cancer xenograft model [38].

Ferritin was also recently employed to develop a novel nanoformulation containing both Rapamycin, an mTOR inhibitor, and Erastin, a ferroptosis inducer. NPs were produced by the emulsification technique, which was shown to be superior compared to the standard pH disassembly–reassembly methodology in regards to drug loading. Interestingly, the size of the NP formed was 7-fold larger than standard FN (78 compared to 12 nm). Nonetheless, this novel formulation achieved impressive results in controlling the tumor growth in a murine model of breast cancer which recapitulates tumor relapse and metastases formation. Briefly, the primary tumor was allowed to grow, and it was excised to simulate surgery. Subsequently, NPs or the free drugs were included in a thermo-responsive F-127 hydrogel and injected into the tumor resection cavity to test the ability of the nanoformulation to prevent tumor recurrence [51]. Unfortunately, the authors did not evaluate the differences in uptake between standard a FN and the modified version developed. Furthermore, since NPs were not administered intravenously, their biodistribution was not evaluated.

FN has also been explored as a nanocarrier for a large variety of drugs for cancer therapy, such as Olaparib [39], Everolimus [40], Curcumin [41], Oxaliplatin + Panitumumab [53], Mertansine [49], Resveratrol [54] and Navitoclax [42].

### 4.1. FN-Based NPs for Immunomodulation and Immunotherapy

Another therapeutic strategy which has recently emerged in cancer therapy is the immunomodulation of the TME, in particular, the reprogramming of tumor associated macrophages (TAMs). In solid tumors, TAMs constitute up to 50% of the tumor mass and have been shown to support local immunosuppression and metastases formation [106]. They are recruited from the bloodstream and surrounding tissues by growth factors and chemokines, including colony-stimulating factor 1, C-C motif ligand 2 and vascular endothelial growth factor [107,108]. Interestingly, TAMs are conventionally categorized as anti-inflammatory M2-like macrophages and express high levels of TfR1 compared to pro-inflammatory M1-like macrophages [109,110]. For this reason, TAMs could be effectively targeted by FN-based therapeutics. Of note, macrophages, in general, can be considered as “gate-keepers” of iron metabolism due to their involvement in the recycling of iron from dying erythrocytes [111]. In addition, TAMs-derived FNs have been shown to function as growth factors on malignant mammary epithelium in a process independent of iron [112]. In order to re-educate TAMs and promote a phenotype switch from M2-like to antitumoral M1-like, FNs have been developed to deliver the toll-like receptor 9 agonist CpG, a nucleic acid with M1-polarizing properties [57]. FNs were functionalized with the TAMs-targeting peptide M2pep (YEQDPWGVKWWY), which was combined with a cationic peptide to allow the attachment of the negatively charged CpG. Interestingly, this novel formulation was able to achieve reduction in tumor growth in a murine model of breast cancer. However, a similar effect was seen even when CpG was absent. The authors hypothesized that the antitumoral effect mediated by the M2pep-modifed FN could be mediated by the intrinsic M1-polarizing activity of the cationic peptide included in the modified FN, since the unmodified FN showed only minor antitumor activity [57].

**Figure 5:**
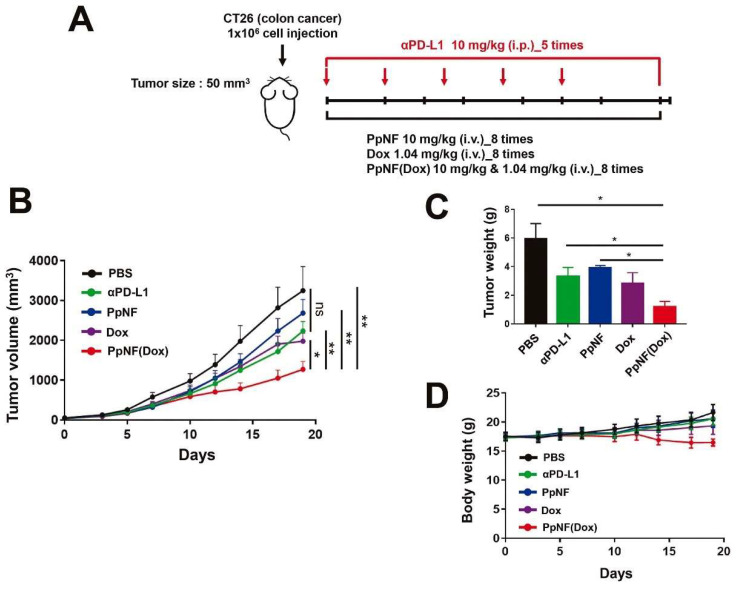
Antitumor activity of FNs displaying the PD-L1 binding peptide (PpNF). (**A**) Experimental schemes for antitumor treatments. Mice bearing s.c. CT26 syngeneic colon tumors were treated with DOX-loaded PpNF (PpNF(Dox)) or PpNF or Dox administered by i.v. injection three times per week. Anti-PD-L1 antibody was administered by i.p. injection twice per week. (**B**) Tumor volumes after treatment. (**C**) The weights of excised tumors from each group at the 19-day post-injection. (**D**) Body weights. The data represent means ± SEM (* *p* < 0.05 and ** *p* < 0.01; *t*-test). Reproduced with permission from [35], Elsevier, 2021.

The discovery of the molecular mechanisms underpinning the immunosuppressive state in the TME led to the FDA approval of immune checkpoint inhibitors (ICIs) for cancer therapy, giving rise to novel immunotherapeutic options that are able to induce a strong infiltration of active immune cells in the TME, with consequent control of tumor growth [113]. ICIs currently used in the clinical setting are monoclonal antibodies (mAbs) that are able to block the activity of the programmed cell death protein 1 (PD-1)/PD-L1 interaction or cytotoxic T-lymphocyte antigen-4 expressed by T cells. Recently, a DOX-loaded engineered FN displaying the PD-L1 binding peptide (PpNF) was developed by Seon and colleagues [34]. Interestingly, this novel nanoformulation was able to achieve enhanced tumor-growth reduction in the colon carcinoma CT26 xenograft model, as compared to anti-PD-L1 mAb and free DOX (Figure 5). In addition, the engineered FNs without DOX were shown to be superior to anti-PD-L1 mAb in enhancing the activity of T cells in vitro.

We hypothesize that, in future years, novel FN-based delivery system will be employed to modulate the activity of immune cells, since a new paradigm for NP-based anticancer therapeutics is emerging [114]. In fact, the expanding arsenal of nanomedicines able to modulate the activity of TME-infiltrating immune cells could be utilized to support standard chemotherapeutics or immunotherapies in order to reactivate the antitumor immunity [115].

### 4.2. FN-Based NPs for the Treatment of Brain Tumors

The blood–brain barrier (BBB) is a diffusion barrier that impedes the influx of most compounds from the bloodstream to the brain parenchyma and represents a protective interface between the central nervous system and peripheral blood circulation [116]. Interestingly, brain cells require iron for metabolic processes; thus, transferrin and ferritin have to bypass the BBB in a process mediated by ligand–receptor recognition. Within the brain, TfR1 was shown to be expressed by capillary endothelial cells, choroid plexus epithelial cells and neurons, which increase the expression of TfR1 in condition of iron deficiency [117]. In addition, TfR1 has been shown to be overexpressed in brain tumors, particularly in glioblastomas, and its overexpression is associated with worse prognosis [118]. Taking into consideration these experimental evidences, we see that FNs are promising candidates for effective brain tumor therapy, due to their intrinsic targeting capability toward TfR1.

Fan and colleagues demonstrated that DOX-loaded FNs are able to bypass the BBB and deliver DOX to brain tumors in mice, dramatically increasing their survival compared to mice treated with control treatments (free DOX and Doxil™) [43]. Interestingly, FNs maintain their intact structure after crossing the BBB by transcytosis. This process is mediated by endothelial cells and allows the accumulation of FNs in the brain parenchyma in healthy mice. However, FNs co-localize with lysosomes after internalization in glioma cells. These results corroborate the idea that FNs traverse the BBB and effectively deliver therapeutics to brain tumors without affecting the surrounding tissues. The authors speculate that the different fate of FNs between endothelial and tumor cells could be due to the differences in expression of TfR1 [43]. More recently, α2β1-targeted Dox-loaded FN (αβ-FN-DOX) was shown to have enhanced activity compared to FN–DOX in controlling the tumor growth of glioblastoma in an orthotopic model of brain tumor (U-87MG) [44]. Interestingly, αβ-FN–DOX had a higher drug-loading capacity compared to FN–DOX (60 vs. 15%, respectively). The authors speculate that this could be due to the modified integrin α2β1 targeting sequence, which possesses multiple carboxyl groups that could have an impact in ionic interactions between DOX and αβ-FN.

In another report, PTX-loaded FNs were successfully developed by the disassembly and reassembly methodology and were used to treat C6 glioma bearing mice. The results showed enhanced activity of FN–PTX compared to the free drug in controlling tumor growth. Furthermore, treatment with FN–PTX showed no apparent signs of toxicity in the heart, liver, spleen, lung and kidneys of treated animals [45].

Other anticancer compounds, such as Au(III) thiosemicarbazone [55] and vincristine [52], have been successfully loaded into FNs and used to treat brain tumors in various murine models of cancer, achieving impressive results in controlling tumor growth. Interestingly, FNs can also facilitate the delivery of therapeutic mAb through the BBB. Our group has developed FNs coupled with Trastuzumab or Cetuximab, two FDA-approved mABs that are able to target the human epidermal growth factor receptor 2 and the epidermal growth factor receptor, respectively [46]. In addition, our group has developed a methodology to specifically study the translocation of FNs (or potentially other nanocarriers) through the BBB. This ex vivo model is based on layers of primary rat brain microvascular endothelial cells and astrocytes, which are used as a surrogate of the BBB [119].

We speculate that the development of novel FN therapeutics for the treatment of brain tumors will be particularly prominent in the coming years, since FNs have shown to effectively bypass the BBB without disassembly, leading to the release of FN-loaded therapeutics directly to tumor cells and avoiding off-site side effects.

## 5. Preclinical Exploitation of FN-Based NPs for Tumor Imaging

There is an ever-growing need for novel imaging agents that are able to effectively identify the presence of very small tumors in the early stage of the pathology, when they can be successfully treated by surgery or anticancer therapies. In addition, after successful surgery, patients undergo routine diagnostic tests to reveal the insurgence of metastatic events that could occur even several years after the original diagnosis [120]. Unfortunately, metastatic tumors are often incurable, since they usually become resistant to standard therapies and account for 90% of total cancer death worldwide [121]. Hence, it is critical to identify the presence of metastases with the current imaging modalities (computerized tomography (CT), magnetic resonance imaging (MRI) and positron emission tomography (PET)) which are often utilized together with contrast agents that are able to accumulate specifically in the TME.

In regards to MRI, gadolinium-based contrast agents are widely used, since they are able to identify highly vascularized tissues, such as tumors [122]. However, these types of agents are not cancer-specific and can result in a high rate of false positives. In addition, standard MRI does not have the sufficient spatial resolution to detect micro-metastases, thus leading to possible misdiagnoses of oligometastatic disease [123]. On the other hand, the glucose analog 18F-Fluorodeoxyglucose is a contrast agent utilized in PET/CT imaging to detect tumors, allowing for the visualization of areas with high metabolic activity [124]. Unfortunately, other areas characterized by active metabolic activity (benign tumors, inflammation sites and areas of ongoing infections) can give rise to false-positive results. Lastly, PET scans are quite expensive and require the use of radioactive contrast agents based on glucose that cannot be utilized in pregnant women or diabetic patients, due to off-target side effects [125].

NP-based delivery systems have been employed to enhance the specificity of contrast agents for tumor cells [126,127,128]. FNs have also been explored as a delivery platform for imaging probes, particularly for metal-based contrast agents. For example, multimodal FNs loaded with superparamagnetic iron oxide (Magnetoferritins) and a near-infrared fluorescence dyes were developed to efficiently detect tumors by multiple imaging modalities [58,59,65]. Interestingly, Magnetoferritins can efficiently identify very small tumors (~1 mm) by MRI in murine models of cancer, thus dramatically increasing the limit of detection of current contrast agents for MRI. Iron-loaded FNs derived from equine spleen (HoS–FN, composed of 85% of Lc and 15% of Hc subunits) were also utilized for MRI visualization of tumors [61]. Interestingly, HoS–FN showed enhanced uptake by SCAR5-positive cells, due to the specific targeting of this receptor mediated by Lc subunits. Furthermore, HoS–FNs were also able to reduce tumor growth, as compared to apoferritin HoS–FN in a murine model of breast cancer.

Imaging agents are also used in fluorescence-guided oncological surgery to assist the surgeon in the identification of metastatic foci, particularly in lymph nodes [129]. Indocyanine-green (ICG) is one of the most used FDA-approved fluorescent dyes for this purpose, since it can be visualized avoiding background autofluorescence (mainly due to hemoglobin) and has a low risk of adverse events [130,131]. Our group has developed ICG-loaded FNs with improved fluorescence accumulation in tumors in comparison to free ICG in a murine model of breast cancer [26,63]. Since specific accumulation of FN–ICG in tumors can be detected up to 24 h after intravenous injection in mice, we speculate that FN–ICG could be administered prior to surgery, and it could be visualized during surgery by fluorescence-guided endoscopy. This methodology could potentially reduce surgery time and improve the detection of small metastases, particularly in lymph nodes.

FNs were also developed to specifically visualize bone metastases by genetic manipulation of ferritin to include osteoblast and hydroxyapatite-binding peptides [64]. In another report, folic acid–functionalized FNs were developed to target tumor cells and deliver perfluoropentane, a compound used for low-intensity focused ultrasound imaging and therapy [62]. Lastly, gold NPs were efficiently encapsulated into 2-amino-2-deoxy-glucose-functionalized FNs to develop a tumor-targeted FN for CT imaging [60].

Overall, FN-based nanostructures can be utilized for the tumor-specific delivery of numerous contrast agents to improve their pharmacokinetic characteristics and enhance tumor accumulation.

## 6. Drawbacks and Future Perspective of FN

FNs are a versatile drug delivery system for chemotherapeutics and imaging agents. However, one of the major limitations of FNs in regards to drug loading is the low encapsulation capacity for hydrophobic compounds. This is primarily due to the leakage of the loaded hydrophobic drug from FN soon after encapsulation. Nonetheless, hydrophobic drugs can still be loaded inside FN by utilizing methodologies such as the pre-complexation with copper ions or the modification of native ferritin with hydrophobic amino acid sequences, that are able to enhance the affinity of hydrophobic compounds for ferritin. For this purpose, Wang and colleagues designed a novel FN construct (Am-PNCage) by linking the sequence of the Pout peptide (GRGDSKKHHHHHHAFAFAFAFVVVAA) to the C terminus of Hc ferritin through a flexible amino acid sequence GGSG, which replaced the E helix amino acids of Hc. This novel FN was employed to achieve the co-loading of the hydrophilic anthracycline EPI and the hydrophobic topoisomerase inhibitor Camptothecin and showed impressive antitumor activity in different murine models of cancer [47]. This novel FN construct could pave the way for the development of sophisticated FN-based nanostructures that are able to integrate multiple drugs with different mechanisms of action. This combinatorial nanotherapy could synergistically strike solid tumors by taking advantage of specific chemosensitivities to limit the insurgence of resistance to single chemotherapeutics.

An important area that is currently understudied is the relevance of the various modifications of FNs in regards to uptake and toxicity, particularly toward immune cells and erythrocytes in the bloodstream. For example, RGD-modified NP (nano-emulsions and liposomes) have been shown to be taken up by phagocytes in the bloodstream that are then able to transport NPs to specific sites in the body where there is ongoing inflammation and/or angiogenesis, such as the TME [132,133]. In fact, a majority of research efforts have been focused on showing that FN-loaded drugs can induce fewer side effects as compared to the free drug. This has been shown extensively for DOX, since FN–DOXs have an encouraging minimal effect on cardiomyocytes when compared to DOX [99,100]. However, it remains unclear if FN modifications can impact their uptake on different cell types present in the bloodstream. This area of study could be particularly relevant to pursue since it has been recently speculated that the enhanced accumulation of nanotherapeutics in the TME could be mediated not only by the EPR effect but also by the phenomenon of NP hitchhiking [81,114,133,134].

Indeed, FNs have favorable and interesting characteristics as an NP-based delivery system. Their efficient loading capacity for different drugs used in oncology, intrinsic targeting toward TfR1 and biocompatibility make them an ideal nano-platform for the treatment and imaging of tumors. Unfortunately, to date, FNs have not yet reached the clinical stage. In fact, the current high cost of production somewhat limits their translational potential. However, we speculate that the recent advancement concerning drug-loading efficiency and customization capabilities could facilitate the interest of pharmaceutical industries in developing novel production protocols for FNs that are aimed at enhancing purity, while, at the same time, reducing the costs of production. Collectively, the number of experimental evidences in support of the use of FNs as nano-delivery systems are ever-increasing, making their translation from bench to bedside a reasonable possibility. Lastly, the opportunity of co-encapsulating different drugs into FNs allows for the development of novel FN-based theranostic agents that are able to combine both imaging and therapeutic functionality in a fully biocompatible nanosystem. For these reasons, we believe that, in the near future, the clinical application of FNs could play a pivotal role in the diagnosis and treatment of solid tumors.

## Figures and Tables

**Table 1:** FN-based NPs for the imaging and treatment of tumors.

FN Origin	Purpose	Modifications	Loaded with	In Vivo Tested?	Reference
Human Hc FN	Cancer therapy	BCP1 peptide	DOX	Yes	[31]
Human Hc FN	Cancer therapy	Mutations to enhance the binding of Cu2+	DOX	Yes	[32]
Human Hc FN	Cancer therapy	4 Lysines (C-terminus)	siRNA (EGFR)	Yes	[33]
Human Hc FN	Cancer therapy	PD-L1 binding peptide	DOX	Yes	[34]
Human Hc FN	Cancer therapy	tLyP-1 peptide	PTX	Yes	[35]
Human Hc FN	Cancer therapy	Trastuzumab	DOX	Yes	[36]
Human Hc FN	Cancer therapy	PEGylation (50% subunits)	DOX	Yes	[37]
Human Hc FN	Cancer therapy	PEGylation (75% subunits)	Acriflavine	Yes	[38]
Human Hc FN	Cancer therapy	None	Olaparib	No	[39]
Human Hc FN	Cancer therapy	None	Everolimus	No	[40]
Human Hc FN	Cancer therapy	None	Curcumin	No	[41]
Human Hc FN	Cancer therapy	Anti FAP antibody	Navitoclax	Yes	[42]
Human Hc FN	Cancer therapy	None	DOX	Yes	[43]
Human Hc FN	Cancer therapy	α2β1 targeting peptide	DOX	Yes	[44]
Human Hc FN	Cancer therapy	None	PTX	Yes	[45]
Human Hc FN	Cancer therapy	Trastuzumab or Cetuximab	Empty	No	[46]
Human Hc FN	Cancer therapy	Pout peptide (C terminus)	EPI, Camptothecin	Yes	[47]
*Pyrococcus furiosus* FN	Cancer therapy	SP94 peptide	DOX	Yes	[48]
Horse spleen FN	Cancer therapy	None	Mertansine	No	[49]
Horse spleen FN	Cancer therapy	None	Arsenoplatin-1	No	[50]
Horse spleen FN	Cancer therapy	Emulsified FN (size 78nm)	Rapamycin and Erastin	Yes	[51]
Horse spleen FN	Cancer therapy	GKRK peptide	Vincristine	Yes	[52]
Unspecified	Cancer therapy	PEG–Panitumumab	Oxaliplatin	Yes	[53]
Unspecified	Cancer therapy	RGD peptide	Resveratrol	Yes	[54]
Unspecified	Cancer therapy	None	Au(III) thiosemicarbazone	Yes	[55]
*Pyrococcus furiosus* FN	Cancer nanovaccine	SpyCatcher	SpyTagged peptides	Yes	[56]
Human Hc FN	Cancer Immunotherapy	M2pep peptide (N-terminus), cationic peptide (C-terminus)	CpG	Yes	[57]
Human Hc FN	Cancer Theranostic	None	Iron Oxide (core) and IRdye800 or DOX	Yes	[58]
Human Hc FN	Cancer Theranostic	Coated with RBC (functionalized with FA)	Iron Oxide, Cy5.5	Yes	[59]
Horse spleen FN	Cancer Theranostic	2-amino-2-deoxy-glucose	Gold NP	No	[60]
Horse spleen FN	Cancer Theranostic	None	Endogenous Iron	Yes	[61]
Unspecified	Cancer Theranostic	PEG–FA	Perfluoropentane	Yes (imaging only)	[62]
Human Hc FN	Tumor Imaging	None	ICG	Yes	[26,63]
Human Hc FN	Tumor Imaging	SDSSD peptide or hydroxyapatite binding peptide	Cy5	Yes	[64]
Human Hc FN	Tumor Imaging	None	Iron Oxide or Cy5.5	Yes	[65]
